# The diverse world within: age-dependent photobiont diversity in the lichen *Protoparmeliopsis muralis*

**DOI:** 10.1093/femsec/fiaf096

**Published:** 2025-09-26

**Authors:** Veronika Kantnerová, Pavel Škaloud

**Affiliations:** Department of Botany, Faculty of Science, Charles University in Prague, Benátská 2, Prague 2, 12800, Czech Republic; Department of Botany, Faculty of Science, Charles University in Prague, Benátská 2, Prague 2, 12800, Czech Republic

**Keywords:** DNA metabarcoding, lichen ontogeny, photobiont diversity, *Protoparmeliopsis muralis*, selectivity, *Trebouxia*

## Abstract

Understanding the initial formation and development of lichens is crucial for elucidating the mechanisms behind the formation of complex lichen thalli and their maintenance in long-term symbioses. These symbiotic relationships provide significant ecological advantages for both partners, expanding their ecological niches and allowing them, in many cases, to overcome extreme environmental conditions. The correct development of thalli likely relies on the selection of suitable photobionts from the environment. In this study, we focused on the impact of lichen age on the overall diversity of photobiont partners and examined how mycobiont preference toward their symbionts changes at different developmental stages. Using the lichen *Protoparmeliopsis muralis* as a model organism, we observed a strong correlation between the diversity of photobionts and lichen age, confirmed by both molecular data and morphological observations. Our findings indicate greater photobiont diversity in older thalli, suggesting that lichens retain the majority of algae they collect throughout their lifespan, potentially as an adaptation to changing environmental conditions. Additionally, we found that some lichen samples contained only low levels of *Trebouxia* algae, indicating that *P. muralis* does not consistently rely on this typical partner and that local environmental conditions may significantly influence its symbiotic composition.

## Teaser

The study reveals that older lichens may host a wider mix of algae, which could help them adapt to environmental changes.

## Introduction

Lichen symbiosis was historically understood as a partnership between two distinct organisms, the mycobiont (fungal partner) and the photobiont (algal or cyanobacterial partner) (Ahmadjian 1987). Today, there is a growing belief that the relationship is predominantly mycobiont-driven, with the photobiont partner acting as a more interchangeable element susceptible to horizontal switching in lichen hosts (Piercey‐Normore and DePriest 2001 , Dal Forno et al. 2021). Despite this dominance, the partnership benefits both participants by enabling adaptation to extreme environments, where neither could survive alone. This symbiosis expands the ecological niche of both the mycobiont and its photobiont, contributing to the widespread occurrence of lichens across various habitats (Ott et al. 1993, Muggia et al. 2013, Peay 2016, Vančurová et al. 2018, Moya et al. 2021).

Lichens host a diverse community of photosynthesizing partners and their distribution in the lichen thallus can be quite heterogeneous. Different compositions have been documented in apical, middle, and basal parts in fruticose lichens (Moya et al. 2017), but also differences in the central and marginal lobes of crustose lichens, with this variability usually assigned to varying microclimatic conditions (Muggia et al. 2014, Noh et al. 2020). The diversity and abundance of individual symbionts within a thallus depend on multiple factors, such as environmental conditions, type of substrate on which the lichen grows factors of the habitat, the lichen age, and the specific lichen biotype, including thallus structure, texture, and reproductive strategies (Fernández‐Mendoza et al. 2011, Marini et al. 2011, Peksa and Škaloud 2011, Leavitt et al. 2016, Steinová et al. 2019, Moya et al. 2021). Furthermore, the symbiotic pair can propagate either together, for example, in the form of asexual propagules such as soredia or isidia, or separately in the form of sexual fungal spores, where no algal partner is present and the photobiont must be found at the new locality and connect de novo in the process of “relichenization” (Pyatt 1973, Beck et al. 1998, Sanders and Lücking 2002). The pool of retrievable algae is also a subject of recent studies as the actual source of algae for each mycobiont can vary significantly, ranging from free living algae in soil or bark, and potentially even from other lichens through overgrowth and subsequent theft of its photobionts (Friedl 1987, Veselá et al. 2024).

A mycobiont can establish a symbiotic relationship with a larger number of photobiont partners over the course of its life, even with suboptimal partners early in thallus development. It has been hypothesized that some mycobionts can replace these initial partners with a preferred photobiont at later stages of development (Friedl 1987, Gaßmann and Ott 2000), ensuring that the dominant photobiont is most favorable for the conditions in which the lichen grows (Piercey-Normore 2004, 2006, Guzow-Krzemińska 2006, Ohmura et al. 2006, Casano et al. 2011, Peksa and Škaloud 2011). The formation of complex lichenized thallus thus likely depends on the principle of partner replacement. In the experimental study by Schaper and Ott (2003), initial thallus formations in the species *Fulgensia bracteata* were observed, first with the optimal partner (the genus *Trebouxia*) and later with a less suitable partner (the genus *Asterochloris*). In both cases, an initial interaction occurred where mycobiont hyphae sought out and tightly enveloped photobiont cells. This new structure was covered by a layer of hydrophobic, protein-rich mucilage resembling accumulated soredia. With the optimal partner, the mucilage envelope then folded into a mucilaginous network, forming the so-called prethallus, which is usually the most advanced developmental stage achieved under laboratory conditions.

On the other hand, in the relationship with the suboptimal partner, lichen development did not progress beyond the soredia-like phase, and overall, the association of both partners was less stable. Thus, it is evident that for a short time at the beginning of development, a mycobiont can form primary associations even with photobiont partners that are not the most suitable for it. However, to form a more complex thallus, it likely needs to replace the initial partner with a more suitable one (Schaper and Ott 2003).

Essentially, all the above studies suggest that in the early stages of lichen development the mycobiont chooses random available algae in proximity, likely trying to survive by entering any possible relationship. Thus, the relichenization process, necessary in the formation of sexually reproducing lichens, is not limited to the association with one specific algal species for each lichen but can for a certain period be formed with any algae, probably to quickly colonize new substrate and easily find the preferred photosynthetic symbionts that can be less abundant in the environment (Ott 1987). There are instances where a lichen can survive with suboptimal partners even for some period and even use saprotrophic or parasitic lifestyle (Gaßmann and Ott 2000). However, the mycobiont will likely need to find a symbiont that is most suitable for it in the later stages to construct a fully developed thallus (Schaper and Ott 2003). Accordingly, we hypothesize that it is probable that a young thallus, although developed, may contain a variety of less favorable photobionts. This greater diversity is likely compared to older thalli, where a single dominant photobiont is usually established (Ott 1987).

The major aim of this study is to test the hypothesis of bigger variability in photobiont choice in the early stages of lichen development compared to older lichen thalli, with size (in terms of area of the thallus) used as a proxy for age determination of the lichen (Armstrong et al. 2015a). We used the lichen species *Protoparmeliopsis muralis* as a model organism, due to its favorable attributes. *Protoparmeliopsis muralis* is a species of terricolous lichens with a foliated, greyish-green thallus. It is a cosmopolitan species that occurs on rocks, tree bark, and many anthropogenic substrates, hence the popular name “urban lichen” (Fałtynowicz 1992). It has been chosen for its well-defined circular thallus, which grows from the center toward the peripheral parts as is common in foliose species (Muggia et al. 2013, Seminara et al. 2018). This provides the advantage of easily identifying each individual. In contrast to other taxa, like *Cladonia*, which primarily produces soredia propagules (Steinová et al. 2019, Černajová and Škaloud 2020), the thalli of *Protoparmeliopsis* reproduce both sexually and asexually, but most often sexually. This increases the potential variability of compatible partners in this genus because the algal partner is typically chosen from the pool of free-living algae at the site where the mycobiont hypha lands (Bowler and Rundel 1975, Rikkinen et al. 2002). Additionally, the plurality of algal photobionts within the genus has already been documented in previous studies (Guzow-Krzemińska 2006, Muggia et al. 2013).

## Materials and methods

### Sampling

More than 100 samples of *P. muralis* were collected at the long-term study site at Vinařická hora in the Czech Republic (50.181684, 14.089558). Lichen thalli were collected from saxicolous substrates according to their size, which is often given as a proxy of the ontogenetic state (or age/developmental stage) of the lichen (García and Rosato 2018, Molins et al. 2021). Samples ranged from the smallest collected thalli with a surface area of around 0.022–1 mm^2^, representing only the initial lobes of the lichen, up to the biggest fully developed thalli, which often contained reproductive structures (apothecia), with a surface area of around 20–117.2 mm^2^ (Table S1).

The collected thalli were photographed using a stereomicroscope Leica S9D with a Flexacam C3 microscope camera and their area was measured using the image processing tool ImageJ v1.54, to correlate data about the photobiont diversity with the respective ontogenetic stage of the individual lichens. The surface of the thalli was washed with distilled water and an alcohol solution (96% ethanol) to ensure clean samples without contamination from other epiphytic algae.

### The morphological algal determination

The Nikon Eclipse 80i fluorescence microscope was used to determine the morphology of the algal symbionts within the photobiont layer of the lichen. Very thin slices of the thalli of *P. muralis* were observed under the fluorescence microscope and photographed using the camera on top.

The structure of the photobiont layer was visualized using the inverted confocal microscope Leica TCS SP2 with an AOBS (Acousto-Optical Beam Splitter) system, which ensures a high degree of sensitivity and combines up to four fluorescent markers. The advantage was the possibility to scan live samples layer by layer of our cut of the thalli. From these layers it was possible to create a composite fluorescent photo of the thallus and create reconstruction of biological structures in ImageJ v1.54. To help us better visualize the individual positions of the cells in the photobiont layer.

### DNA isolation, PCR, and DNA metabarcoding

For the smallest lichens, the whole thalli were used and for the larger lichens, different parts of the thalli were collected and included in one sample to ensure that if the distribution of different algal genotypes in the thalli was not homogeneous, the full diversity was still included. Samples were homogenized and used for DNA extraction. Lichen samples of different sizes were isolated by following the CTAB extraction protocol (Cubero et al. 1999). Both algal and fungal DNA were PCR (Polymerase Chain Reaction) amplified to distinguish the main (most abundant) symbiotic partners. The fungal ITS (Internal Transcribed Spacer) region was amplified to confirm the correct morphological determination of the host lichen species (especially for distinguishing the smallest lichen thalli), using Ascomycete-specific forward primer ITS1F (5′-CTT GGT CAT TTA GAG GAA GTA A-3′) (Gardes and Bruns 1993) and the fungal universal reverse primer ITS4 (5′-TCC TCC GCT TAT TGA TAT GC-3′) (White et al. 1990 ). The algal ITS rRNA was amplified using the algal-specific nr-SSU-1780 primer (5′-CTGCGGAAGGATCATTGATTC-3′) (Piercey‐Normore and DePriest 2001) and the universal reverse primer ITS4 (5′-TCC TCC GCT TAT TGA TAT GC-3′).

PCR amplification was conducted in a volume of 20 µl, which consisted of 14.2 µl of water, 4 µl of My Taq PCR buffer (Sigma), 0.3 µl of forward and 0.3 µl of reverse primer, and 0.2 µl of My Taq DNA polymerase (Sigma) with the addition of 1 µl of sample DNA. The PCR reaction followed these steps: initial denaturation at 94°C for 1 min followed by 35 cycles of denaturing at 94°C for 45 s, annealing at 60°C for 1 min, and elongation at 72°C for 2 min, with a final extension step at 72°C for 10 min for the algal ITS rRNA region.

The PCR product was retrieved on a 1% agarose gel with ethidium bromide staining using the process of electrophoresis. The final PCR products were purified using the Left Side Size Selection process with the Agencourt AMPure XP Magnetic Beads (Beckman Coulter). The purified PCR products were Sanger sequenced using the same primers—mentioned above, at Macrogen in Amsterdam, Netherlands.

To uncover the overall diversity of the photobiont partners, we employed DNA metabarcoding. There was no difference between the sequencing depth for younger or older thalli. The algal ITS rRNA was amplified using the newly designed forward primer 1378j02 (5′-TTG CCT TGT CAG GTT GAT TCC-3′) and the universal reverse primer ITS4 (5′-TCC TCC GCT TAT TGA TAT GC-3′) in the first step and barcoded 5.8F-Chlorophyta (Vančurová et al. 2020a) and ITS4 primers in the second step. The PCRs were conducted in a volume of 20 µl, consisting of 10 µl of Q5^®^ High-Fidelity DNA Polymerase, 1 µl each of forward and of reverse primers, with the remaining volume supplemented by water and sample DNA, selected according to the DNA concentration of the samples. In the first step, the PCR conditions were: an initial denaturation at 98°C for 30 s followed by 24 cycles of denaturing at 98°C for 10 s, annealing at 52°C for 45 s, and elongation at 72°C for 1 min, with a final extension step at 72°C for 2 min. In the second step, conditions were: an initial denaturation at 98°C for 30 s followed by 22 cycles of denaturing at 98°C for 10 s, annealing at 52°C for 45 s, and elongation at 72°C for 1 min, with a final extension step at 72°C for 2 min. Each sample was run in two replicates, and we further included 20 PCR negative controls (with distilled water as the template) and 20 multiplexing controls (unused combinations of left and right barcodes). The final PCR products were evaluated using electrophoresis on a 1% agarose gel with ethidium bromide staining, purified using Left Side Size Selection process as specified above, pooled equimolarly, and sent for library preparation and sequencing to Fasteris (Plan-les-Ouates, Switzerland). Sequencing was performed on the Illumina MiSeq platform with paired-end mode (2 × 300 bp).

### Sequence data processing

Sanger sequences were analysed and assembled using the SeqAssem program (SequentiX Software). The alignments of selected sequences were created using MAFFT v. 7.526 software (Katoh and Standley 2013) with the G-INS-I strategy and manually edited in MEGA v. 6.0 (Tamura et al. 2013). Reference sequences were added from GenBank to cover all known lineages in both *Trebouxia* and *Protoparmeliopsis*. To identify *Trebouxia* species in this study, BLAST searches were performed using all newly generated sequences to determine their closest matches in the overall database. A comprehensive dataset was compiled by incorporating reference sequences from the works of Muggia et al. (2020), Leavitt et al. (2015), and Xu et al. (2020). Based on this collection, a phylogenetic tree was constructed including both the reference sequences and our new sequences. To enhance the clarity and precision of the phylogeny, sequences that did not closely resemble our new ones were filtered out (based on percentage of identity), and only those with high similarity were retained. Since BLAST hits can be unreliable and many *Trebouxia* species in public databases are misidentified, we consistently referred to the *Trebouxia* Research Portal (https://trebouxia.net/; currently the most up-to-date *Trebouxia* database) for clade designations. These were assigned following the OTU (Operational Taxonomic Unit) tables provided on the portal.

The phylogenetic trees were inferred by maximum likelihood (ML) analyses in IQ-TREE v. 2.3.6 (Minh et al. 2020) and the best-fit model was selected by ModelFinder using BIC (Bayesian Information Criterion) (Kalyaanamoorthy et al. 2017), using the SYM+I+G4 substitution model. The ML bootstrap support values were calculated based on 1000 replications.

The Illumina MiSeq pair-end reads were processed according to Bálint et al. (2014), including quality filtering, pair-end assembly, removal of primer artifacts, extraction of reads from different lichen thalli using left and right barcodes, reorientation of reads to 5′–3′, demultiplexing, dereplication, and chimera filtering. The clustering was carried out using Swarm v. 2 (Mahé et al. 2015), with denoising set to d = 3, generating a total of 6399 denoised amplicons (swarms). Unlike traditional OTU clustering, which applies fixed similarity thresholds (e.g. 97%), Swarm utilizes an iterative single-linkage approach that enables precise, locally adjusted clustering, avoiding arbitrary cutoffs. The choice of Swarm provides a balance between the broad grouping of traditional OTUs and the fine-scale resolution of amplicon sequence variants (ASVs) representing exact sequence variants without clustering (Fasolo et al. 2024). To avoid spurious sequences, only swarms found in at least 10 reads and in both replicates were considered. The swarms were identified by BLAST searches in SEED2 (Větrovský et al. 2018), using the remote BLAST searches. Only green algal nonchimeric sequences were further processed, i.e. a total of 137 swarms (Table S2, first list). Of these, 30 were present in negative controls in at least three reads. For these swarms, the highest abundance in any negative control was subtracted from their abundance in each sample to mitigate contamination effects (Davis et al. 2018). Afterwards, the replicates were combined, and the higher abundance from either replicate was selected for each sample. Finally, all swarms were aligned using MAFFT and a ML phylogenetic tree was constructed as described above. Every swarm was then visually inspected to identify chimeric sequences and pseudogenes not detected by automatic chimera detection. After removing chimeric sequences and pseudogenes, the final phylogenetic tree was inferred as described above. Finally, all swarms belonging to the same phylogenetically defined species were merged and their abundance was summed (Table S2, second list). To account for the variable number of reads, each sample was normalized to the 0.5th percentile of the most abundant sample (1 183 613 reads) using the “rarefy_even_depth” function in the phyloseq package (McMurdie and Holmes 2013), so that each sample was standardized to contain 5918 reads. Nine samples with lower abundance were normalized to 5918 reads (Table S2, third list).

### Data analysis

All swarms were carefully phylogenetically determined into species as described above. Barplots of relative species abundances were created in R, using the “ggplot2,” “reshape2,” and “funrar” packages (Wickham 2007, 2016, Grenié et al. 2017). Several regression analyses were conducted to test the relationship between the thalli size and diversity patterns, including number of species, number and abundance of symbiotic species, abundance of zoosporine and autosporine species, and abundance of non-*Trebouxia* species. To define the traits mentioned above (autosporine, zoosporine, and symbiotic algae) we used information from the Syllabus der Boden-, Luft-, und Flechtenalgen (Ettl and Gärtner 1995). All tests were conducted in R v.4.4.2 (R Core Team 2023. Accessed October 3, 2025).

## Results

### Sanger sequencing of the mycobiont and photobiont

The identity of the *P. muralis* was genetically confirmed by Sanger sequencing (Table S2). Even the smallest thalli were collected correctly, which gives a good idea about the visual differentiation of this species and the possible easy identification *in situ*, even of the smallest growth stages. We collected over 100 individual lichen thalli samples, in approximately equal quantities across three size categories. After careful examination to exclude thalli exhibiting visible surface damage or fungal overgrowth, a total of 79 samples were selected for sequencing: 22 thalli of the smallest size, with a surface area up to 1 mm^2^, 35 thalli were of the intermediate size (area 1–10 mm^2^), and the remaining 22 thali included fully developed thalli, with a surface area higher than 10 mm^2^. Out of these, we were able to obtain 48 algal sequences that were later analysed and fitted onto a phylogenetic tree, and in this way we confirmed the genus *Trebouxia* as the most dominant photobiont, corroborating previous studies (Sanders and Lücking 2002, Guzow-Krzemińska 2006). All the sequences found using Sanger sequencing belonged to the clade A of *Trebouxia* (Muggia et al. 2020) and the most common species found were *T. incrustata, T. vagua* (including both A04 and A10 OTUs), *T. asymmetrica, T. decolorans, T. arboricola*, and *T. cretacea* (Fig. 2). Interestingly there were also sequences of other genera found in the smallest thalli, such as *Pseudochlorella pyrenoidosa* and *Asterochloris magna*, but these were only in two samples, hence it was not conclusive enough.

### DNA metabarcoding

**Figure 1.**
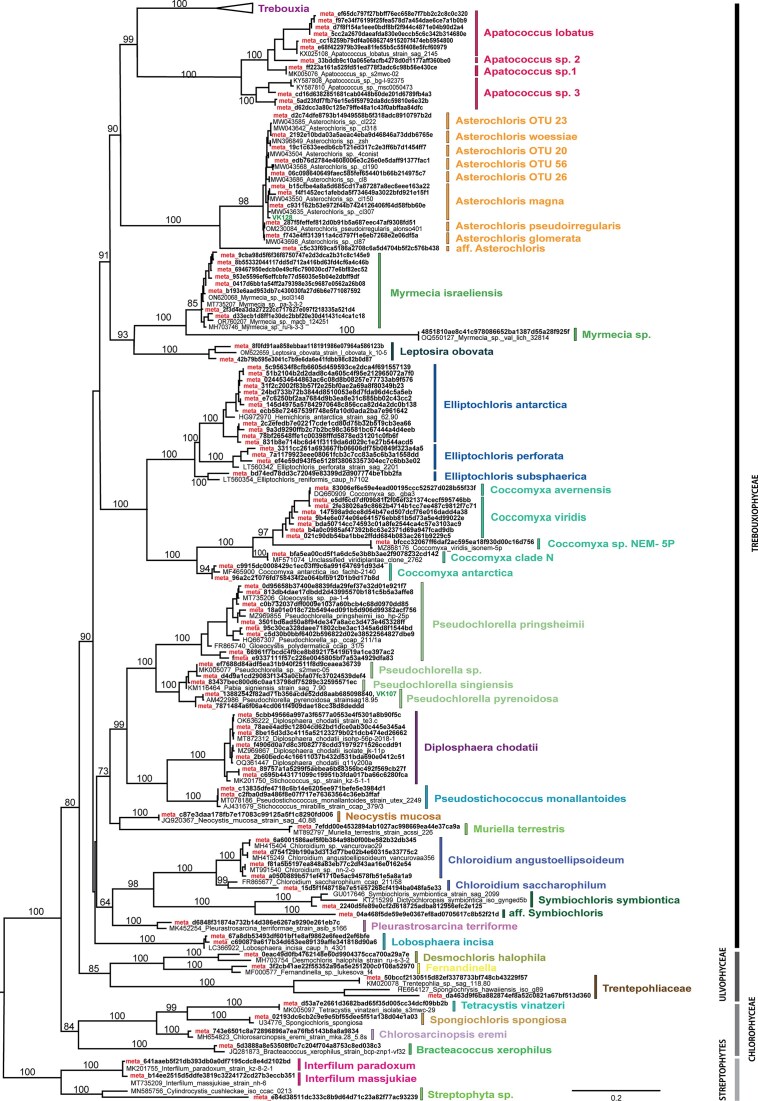
The phylogenetic tree of ITS sequence data for the complex diversity of photobionts within the lichen *P. muralis* generated by IQ-TREE analyses. Only the bootstrap values over 60% are given. The tree is rooted by Streptophyta lineages and the new sequences obtained in this study are marked in bold. DNA metabarcoding sequences are supplemented with "meta" prefix and identified by hashing codes, whereas two Sanger sequences (other than *Trebouxia* species) are identified with VK as abbreviation. The affiliations to algal classes are suggested on the right side.

**Figure 2.**
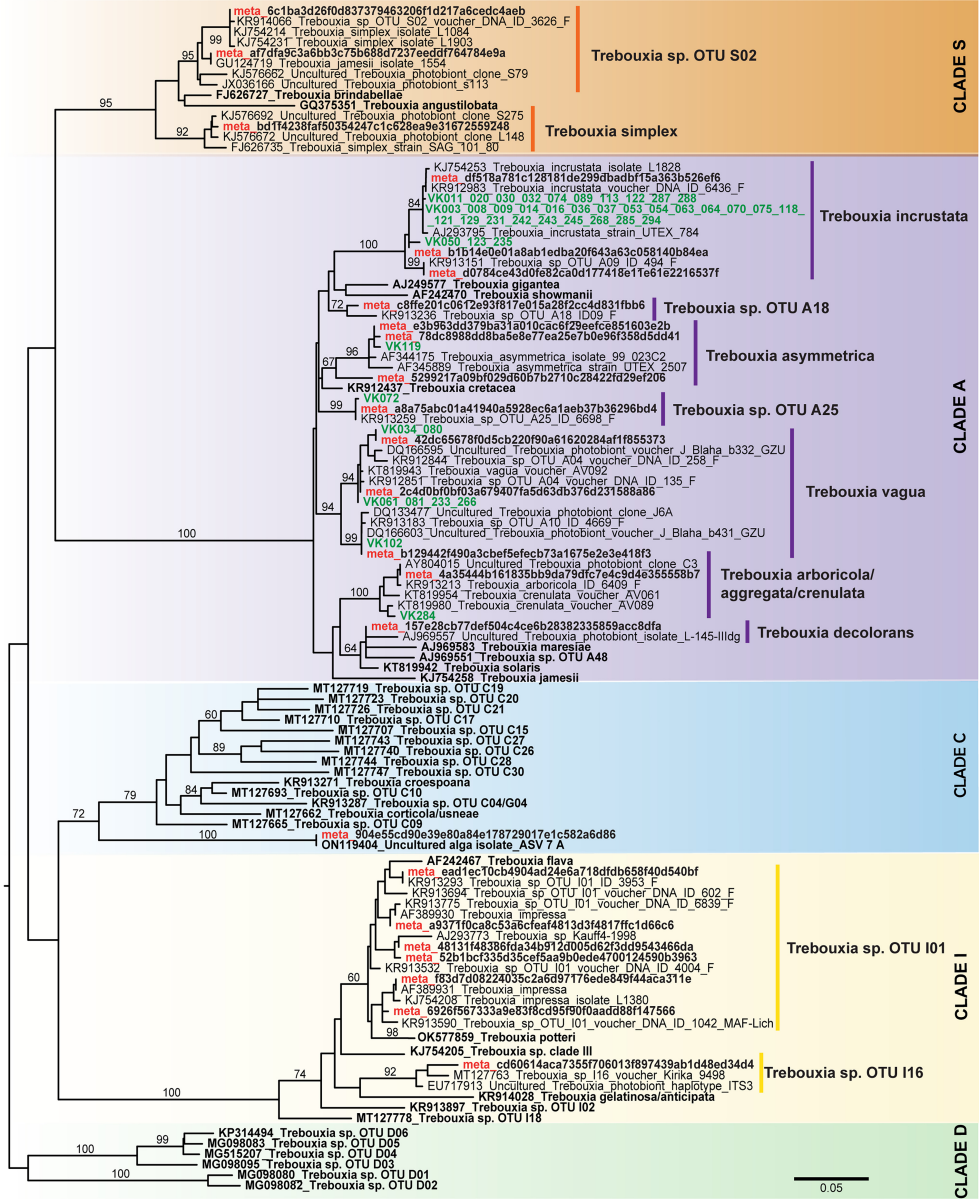
The phylogenetic tree of ITS sequence data of the genus *Trebouxia* generated by the IQ-TREE analyses. Only the bootstrap values >60% are given. The tree is rooted at the *Trebouxia* clade D and new sequences obtained in this study are marked in bold. DNA metabarcoding sequences are supplemented with "meta" prefix and identified by hashing codes, whereas the Sanger sequences are identified by VK as abbreviation.

The overall diversity of photobionts within the thallus was assessed using DNA metabarcoding. We acquired a total of 3 330 802 green algal reads, and from this it was possible to identify 64 distinct species of algae, 43 of which were previously described as photobionts of lichens. These were mostly represented by species from the class Trebouxiophyceae (∼77%), followed by species belonging to the classes Chlorophyceae, Ulvophyceae, and the clade Streptophyta (Fig. 1, Table S2). From the most common genus *Trebouxia*, we distinguished 12 different species belonging to 3 clades, with the most common overall being *T. incrustata* (Fig. 2). The most sequences were from the clade A (*arboricola*/*gigantea*-type), then clade I (*impressa/gelatinosa*-type), and clade S (*simplex/jamesii*-type) (Muggia et al. 2020). We were also able to obtain one *Trebouxia* sequence that belongs somewhere at the base of the clade C (*corticola*-type), which was only once before described by Dreyling et al. (2022), as an uncultured alga isolate ASV_7_A.

### Correlation between lichen size and algal abundance

**Figure 3.**
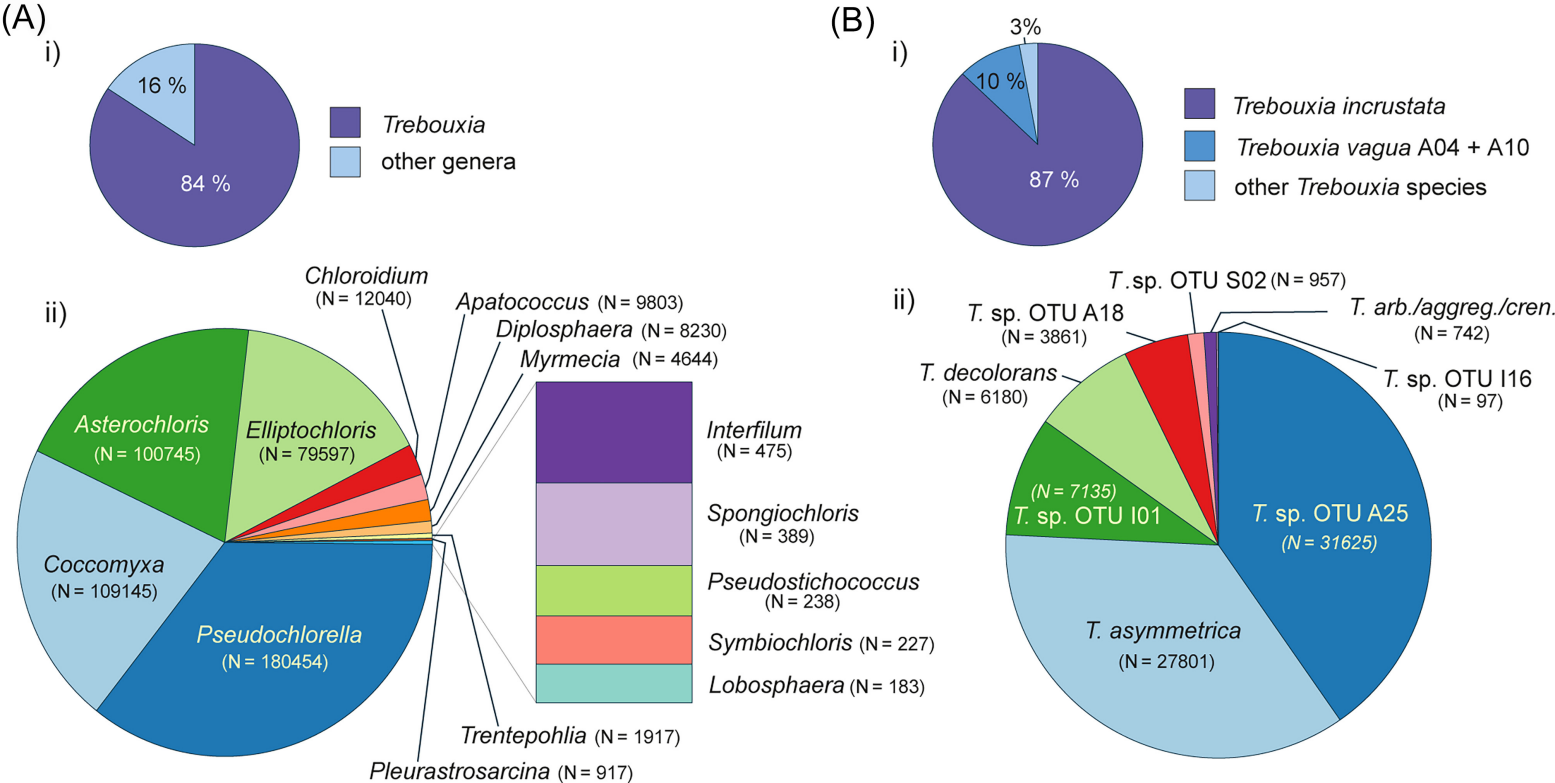
(A and B) The abundance of the algae within lichen *P. muralis*. (A) Two pie charts showing the abundance of the most common algal genera in the thallus: (i) the percentual representation of the most common genus *Trebouxia* compared to all other abundant genera and (ii) abundance of most common genera, excl. genus *Trebouxia*. (B) Two pie charts showing the abundance of *Trebouxia* species: (i) the percentual representation of two most abundant *Trebouxia species*—*T. incrustata* and *T. vagua* (including both A04 and A10 OTUs) compared to all the other species and (ii) abundance of most common *Trebouxia* species without *T. incrustata* and *T. vagua* (both A04 and A10 OTUs) (with largest number of reads acquired), *N* = number of reads.

**Figure 4.**
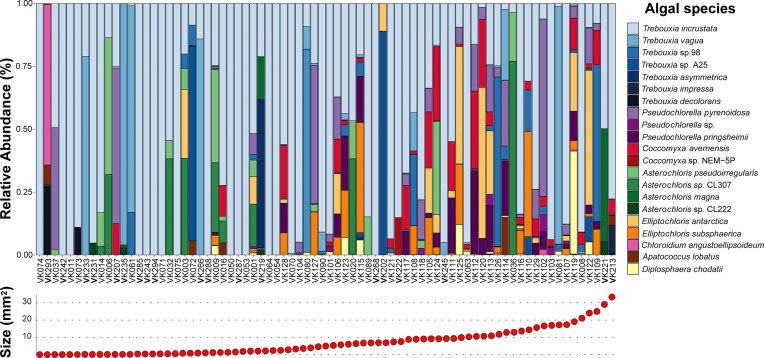
The relative abundance (%) of different photobiont species within the lichen *P. muralis*. Only the most abundant species are included. On the *x*-axis are different lichen samples ranging from the smallest in area on the left up to the largest on the right (marked by the plot in the lower part of the graph). Different colors on the plot correspond to the species list on the right side and the color hue always corresponds to a certain genus. The graph shows the increasing diversity in the larger/older lichen thalli.

**Figure 5.**
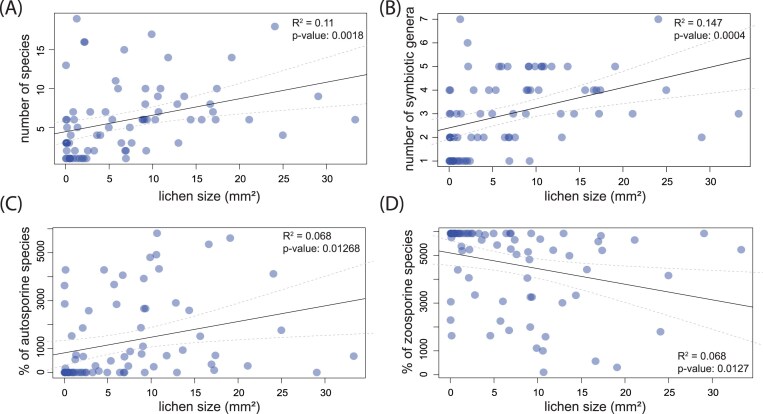
The regression analyses showing the dependence of algal diversity (abundance) on lichen size (the age of the lichen). (A) All species found, (B) only photobiont genera, (C) abundance of autosporine species of algae, and (D) abundance of zoosporine species of algae. They show significant influence of lichen size (age) on the abundance of algal symbionts in lichen *P. muralis*.

The most abundant genera found were *Trebouxia* (comprising ~81.6% of reads), *Pseudochlorella, Coccomyxa, Asterochloris, Elliptochloris, Chloroidium, Apatococcus*, and *Diplosphaera* (Fig. 3A). Among the *Trebouxia* species, the most common were *T. incrustata* and *T. vagua* (including both A04 and A10 OTUs and accounting for ~97% of all *Trebouxia* species), both from clade A. Compared to these, the abundance of other species rapidly decreases within the thallus (Fig. 3B). Smaller quantities also included the genera *Myrmecia, Symbiochloris, Lobosphaera, Apatococcus, Muriella, Pleurastrosarcina, Spongiochloris, Pseudostichococcus, Leptosira, Chlorosarcinopsis, Spongiochrysis, Fernandinella, Tetracystis, Bracteacoccus, Desmochloris, Neocystis*, and *Interfilum*. We observed a noticeable variation in the relative abundance of algal species as the size of the lichen thalli increases, suggesting a complex and varied symbiotic relationship (Fig. 4). While *T. incrustata* was dominant across a range of thalli sizes, it was particularly prevalent in smaller thalli, where it was frequently the only algal species detected. Although we did not notice any species preference for smaller thalli, certain taxa, such as *Elliptochloris perforata, Asterochloris glomerata*, and *Asterochloris* sp. CL8 were detected only in larger thalli (i.e. those with an area larger than 6 mm^2^). Indeed, the diversity in smaller (younger) lichen thalli was significantly lower than in older thalli (Fig. 5A). When focusing solely on symbiotic genera, the proportion of explained variability increased (Fig. 5B), suggesting a more distinct pattern, favoring symbionts.

In 10 samples, the genus *Trebouxia* was present at very low abundance, ranging from ~2% to a maximum of 26% of the total algal reads within the thallus (VK120 = 2%, VK036 = 4%, VK119 = 4.6%, VK125 = 8.3%, VK102 = 9.8%, VK006 = 14%, VK122 = 16.6%, VK112 = 17.7%, VK124 = 18.2%, and VK293 = 26%). This indicates that *Trebouxia* is not consistently the dominant algal genus associated with *P. muralis*. In these samples, the most abundant genera were instead *Asterochloris, Pseudochlorella, Elliptochloris*, or *Chloroidium*.

Finally, we found that thallus diversity varied according to the algal partner’s reproduction strategy (Fig. 5C and D). As the lichen aged, autosporine algae became more prevalent at the expense of zoosporine algae, which were significantly more common within young thalli.

### Morphological observations

**Figure 6.**
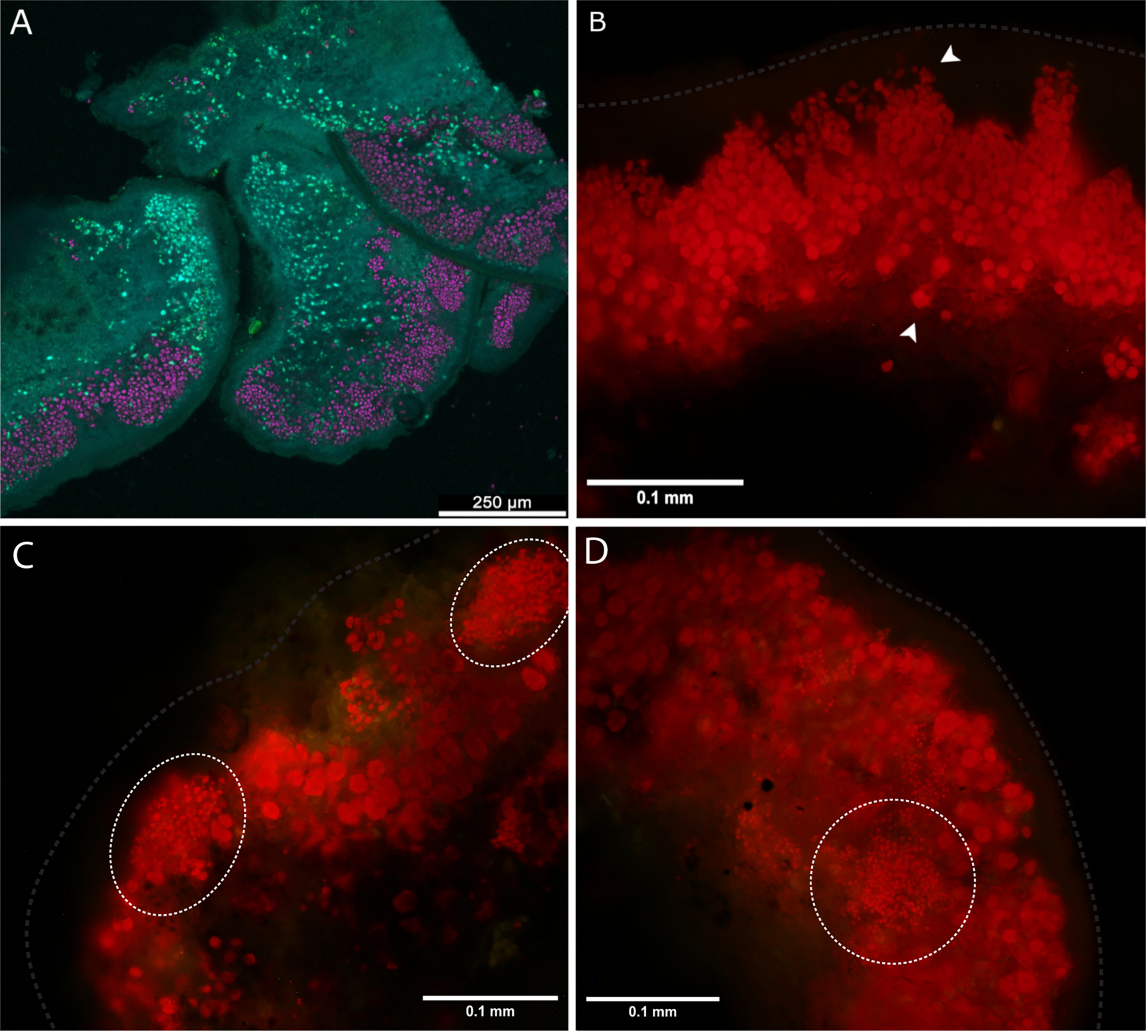
The confocal (A) and fluorescence (B–D) visualization of the photobiont layer within the lichen *P. muralis*, (A) overall view of the photobiont layer inside a thallus lobe of *P. muralis* visible in purple color, in green are the dead algal cells and amorph crystals within mycobiont hyphae, (B) white arrows mark smaller cells of *Trebouxia* situated closer to the cortex and bigger cells concentrated lower, closer to the medulla of mycobiont hyphae, visible in red autofluorescence, and (C–D) white dashed circles highlight *Coccomyxa* cells distributed within the layer of *Trebouxia* algae. A dashed light grey line outlines the approximate position of the upper cortex of *P. muralis*. Scale bars: (A) 250 µm, (B, C, and D) 0.1 mm.

The algal layer was usually not completely uniform, but created rather palisaded structure, where the cell layer of photobionts is thicker in certain places and thinner in between. In many places it was also visible that the cell size decreased toward the cortex layer, suggesting that the algae were probably spatially limited there or that they were much more exposed to light and therefore under more stress than algae in the lower layers (Fig. 6A and B).

**Figure 7.**
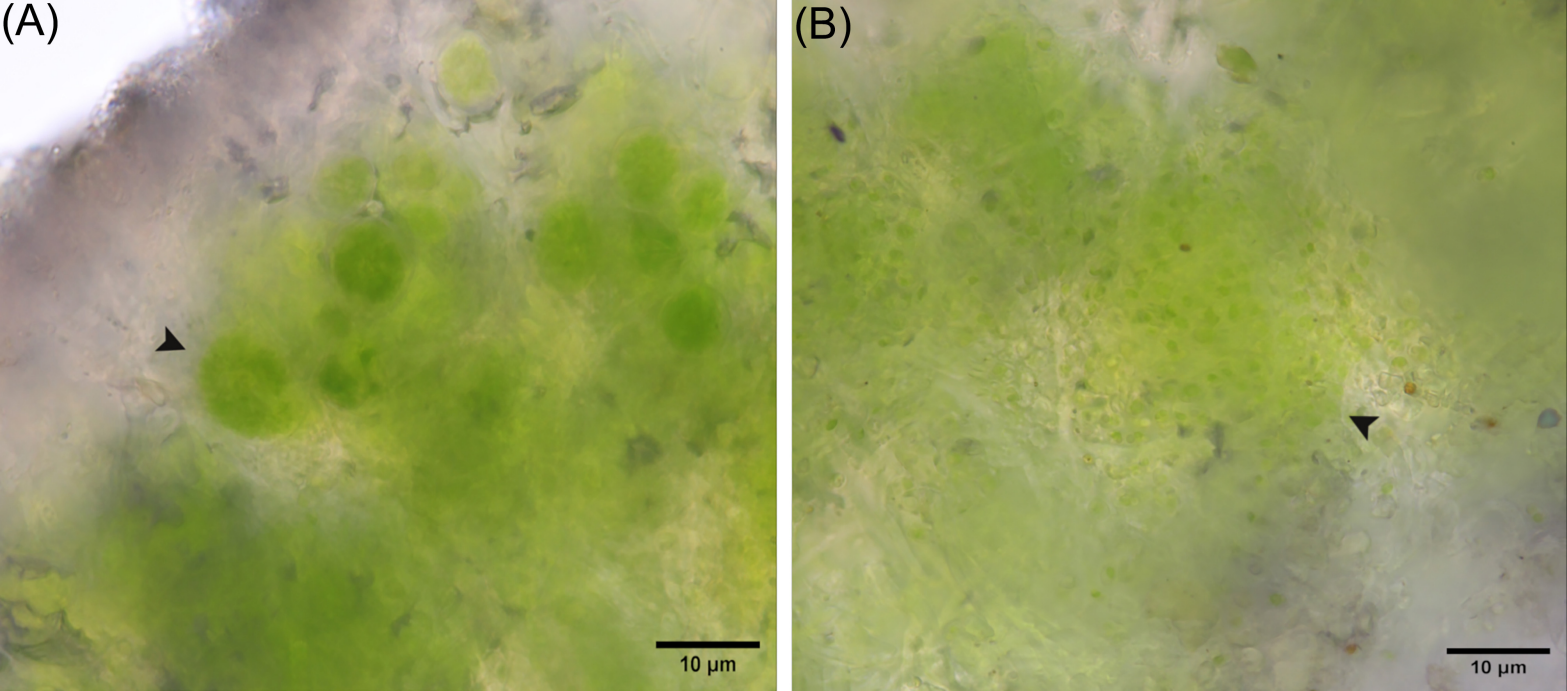
The light microscopy visualization of the photobiont layer, showing two different algal genera embedded within the mycobiont hyphae. (A) The much bigger coccoid cells of the genus *Trebouxia* with central pyrenoid and star-like morphology of the chloroplast and (B) smaller slightly elongated cells of the genus *Coccomyxa* with parietal chloroplast and no pyrenoid, creating colonial clumps. Scale bars: 10 µm.

The plurality of photobionts was not only confirmed by DNA metabarcoding, but we were also able to visualize the presence of different algal genera within the thallus photobiont layer microscopically (Figs 6C, D, and 7). Although we were not able to distinguish the exact species of photobionts present by using this method, our results support the data obtained by the sequencing, morphologically confirming the presence of multiple genera differing by cell size and structure. The most common genus found and easiest to recognize by morphology was *Trebouxia*, which was visible in all cuts of thalli, but we also noted the presence of cells from the genus *Coccomyxa*. From the 100 samples used for DNA metabarcoding, we obtained *Coccomyxa* sequences in 37 samples. Out of the ~50 cross-sections of the other thalli collected for morphology observations, we found *Coccomyxa* cells inside the photobiont layer in two cases (Fig. 6C and D). We visualized the clear cooccurrence of the genera *Trebouxia* and *Coccomyxa*, with the cells of *Trebouxia* being the larger ones with stellate chloroplasts, quite large pyrenoids visible in the cell centre and being the main part of the photobiont layer (Fig. 7A). On the other hand, *Coccomyxa* cells were much smaller, with parietal chloroplasts lacking pyrenoid, forming compact colonial clumps, probably with a thick gelatinous cover. It was evident that the cells of *Coccomyxa* were encircled by the mycobiont hyphae, hence excluding the possibility of these cells being only epiphytic contamination (Fig. 7B).

## Discussion

### The immense diversity of algal photobionts within lichen thalli

Recent studies, including our own, continue to reveal that the diversity of algal photobionts within lichen thalli is far greater than previously assumed. Molecular data have demonstrated that a single lichen thallus can host multiple algal lineages, often from distinct genera, highlighting the complexity of these symbiotic systems (Blaha et al. 2006, Skaloud and Peksa 2010, Muggia et al. 2013, Moya et al. 2017, Pino-Bodas and Stenroos 2021). So far, this phenomenon has been documented in ~6 cyanobacterial and 20 green algal genera (Dědková et al. 2025).

One of the challenges in studying this diversity is that morphological traits of the lichen thallus do not reliably reflect the internal variability of photobiont associations. Lichens with nearly identical morphology can harbor distinct photobiont lineages (Yahr et al. 2004, Lindgren et al. 2020). While rare, there are cases where photobiont identity does influence thallus morphology. For example, *Lecanographa amylacea* can associate with either *Trebouxia* or *Trentepohlia*, two phylogenetically quite distinct genera, resulting in visibly different morphotypes (Ertz et al. 2018). However, such cases are exceptions rather than the rule, typically involving associations with both green algae and cyanobacteria (Magain et al. 2012).

Conversely, some lichen genera, such as *Cladonia*, include morphologically distinct species that share identical photobionts, suggesting that photobiont identity alone may not always drive morphological differentiation (Steinová et al. 2022). Even at the microscopic level, distinguishing algal species based on morphology is difficult, as the symbiotic state often constrains algal growth and form (Marton and Galun 1976, Elshobary et al. 2015, Satyanarayana et al. 2019). These limitations highlight the importance of molecular tools, such as high-throughput sequencing (HTS), for accurately assessing photobiont diversity.

Understanding this diversity is not merely academic—it has ecological implications. Different photobionts exhibit varying environmental tolerances and ecological niches, even within a single algal genus (Nelsen et al. 2021, Cordeiro et al. 2005, Blaha et al. 2006, Muggia et al. 2014). In the case of the genus *Trebouxia*, species from clade I are typically associated with warmer climates (Molins et al. 2021, Nelsen et al. 2021, Meyer et al. 2023), while members of the *T. simplex* complex thrive in colder, mountainous environments (Muggia et al. 2008, Chen et al. 2024). Interestingly, our study found that *P. muralis* can simultaneously host *T. simplex* along with two clade I species, suggesting that photobiont diversity within a single thallus may serve as a strategy for coping with environmental variability. Lichens can be broadly categorized into two main groups based on their photobiont specificity. Generalist species associate with a wide range of photobionts and are typically found in diverse habitats worldwide, while specialists form more exclusive partnerships, which may limit their ecological range (Helms et al. 2001, Yahr et al. 2004, 2006). Generalists often reproduce sexually, promoting new symbiotic combinations and increasing photobiont diversity (Beck et al. 2002, Cao et al. 2015, Steinová et al. 2019). In contrast, specialists typically reproduce asexually via reproductive organs that contain both symbionts, preserving established symbiotic pairs and resulting in lower photobiont variability (Werth and Scheidegger 2012, Widmer et al. 2012, Steinová et al. 2019). However, there are exceptions to this rule. For instance, a study on *Cladonia* species suggests that even sexually reproducing lichens can exhibit high specificity in their photobiont associations (Steinová et al. 2022), indicating that these relationships are more complex than previously thought.

In our study, we discovered that the photobiont layer in lichens includes not only species from the genus *Trebouxia*, but also many others, as evidenced by the numerous reads obtained through DNA metabarcoding. Additionally, for example, the presence of *Coccomyxa* was also confirmed by morphological observations using fluorescence and light microscopy. Similar genera, typically associated with other lichen species, were previously considered epithalline taxa and presented more as contaminants rather than potential symbionts (Muggia et al. 2013). However, recent research increasingly supports the contrary: rather than being exceptions, such taxa are commonly present in minor proportions, contributing to a complex phycobiome within the lichen thallus. In addition to our study, which supports the presence of genera such as *Coccomyxa*, genera like *Diplosphaera, Pseudostichococcus, Elliptochloris, Stichococcus*, and others are frequently detected through HTS analyses. These findings reinforce our observations and suggest that these algae are not merely surface epiphytes, but integral components of the lichen’s photosynthetic layer (Noh et al. 2020, Vančurová et al. 2020, Chiva et al.^2021^ , ^2022^, ^2023^). While HTS provides valuable insights into the overall diversity, the morphological observations, such as those presented in this study and referenced works, remain crucial for the reliable confirmation of these phenomena. Fluorescence and light microscopy have proven effective for identifying *Coccomyxa* and may also be applicable to other morphologically distinctive genera. However, for more accurate identification, other techniques, such as transmission electron microscopy or microscopy of isolated axenic cultures of symbiotic algae are recommended (Molins et al.^2018^ , Chiva et al. 2021). These approaches are often challenging, since many of these algal species occur in very low abundance and are therefore difficult to isolate or identify in standard lichen thallus sections.

### Photobiont diversity across lichen ontogeny

The relationship between lichen thalli age and photobiont diversity has been sparsely studied, largely due to the difficulty of analysing each growth stage in a single individual. In this study, thallus size (measured in terms of area) was used as a proxy for identification of the developmental stage. In lichenology, particularly under field conditions, size remains one of the most reliable indicators of age due to the typically slow and steady growth rates of lichen thalli (Armstrong et al. 2015a). While thallus size provides an accessible measure of developmental stage and we use it in this study synonymously with age (because in our analyses it served as a direct proxy), age may exert a deeper influence on photobiont diversity. Over time, older lichen individuals are exposed to more environmental fluctuations, colonization opportunities, and symbiotic turnover. This temporal dimension fosters cumulative ecological interactions, potentially leading to a more complex and diverse assemblage of photobionts, which can be very specific for different individuals of lichens and the effects of the environment on them specifically (Armstrong et al. 2015b). In contrast, size alone, though correlated, may not capture the full history of biotic exchange and succession. Therefore, lichen age may play a more pivotal role in shaping photobiont diversity than physical size *per se*. We also believe that the lichen acts differently when at the beginning of the development compared to fully developed and established thallus, which is something that is probably also not a function of size alone.

We observed that photobiont diversity in the lichen *P. muralis* was surprisingly greater in older thalli than in younger ones. This contrasts with findings by Molins et al. (2021), who reported a decline in photobiont diversity with age in the fruticose lichen species *Ramalina farinacea*. Several key ecological and biological factors likely underpin these differences. First, *P. muralis* and *R. farinacea* differ markedly in their reproductive strategies—the former primarily reproduces via apothecia (sexual reproduction), while the latter relies heavily on soredia (asexual reproduction). These strategies influence how photobionts are transmitted and maintained within the thallus. Sexual reproduction often involves the acquisition of new photobionts from the environment, potentially increasing diversity over time, whereas asexual reproduction tends to preserve existing symbiotic combinations, possibly leading to reduced diversity in older individuals. Second, the growth forms of the two species—crustose in *P. muralis* versus fruticose in *R. farinacea*—may also play a role. Crustose lichens are more tightly integrated with their substrate and may experience different microenvironmental conditions and colonization dynamics compared to the more exposed and branched fruticose forms. Third, the geographic and ecological contexts of the two studies differ. Molins et al. (2021) focused on Mediterranean populations, while our study was conducted in temperate regions. Environmental variability, including temperature and moisture regimes, likely influences photobiont availability and selection pressures, contributing to the observed differences in diversity patterns (Fernandéz-Mendosa et al. 2011 , Peksa and Škaloud 2011, Moya et al. 2021).

In addition, the species–area relationship, a fundamental ecological principle, may provide insight into the observed photobiont diversity patterns (Gheza et al. 2023). Larger islands and landscapes tend to support greater species richness due to increased habitat heterogeneity (Connor and McCoy 1979, Hortal et al. 2009). However, while traditional species–area relationships involve passive accumulation of species in larger geographic spaces, the situation in lichens is distinct. The thallus is not merely an expanding substrate—it is a living host that actively interacts with its photobionts, likely maintaining and regulating their diversity over time. A more apt analogy can be drawn with the human microbiome, where microbial diversity fluctuates throughout life. Infants initially harbor a relatively simple microbiome, which becomes more diverse as exposure to external microbes increases with age (Rotimi and Duerden 1981, Beller et al. 2021). Likewise, younger *P. muralis* thalli were found to contain a higher number of rare algal species.

The timing of when lichens acquire additional taxa varies and is not well understood. Most are likely obtained early in development when the thallus is not yet fully formed, making it easier for algae to become trapped. However, our data show that some taxa are acquired later in the lichen development, contributing to the greater diversity observed in older thalli. This supports studies such as the study of foliicolous lichens, where specialized “prothallus” is formed as a new structure on already existing fully developed thallus. It is a part of the lichen that can grow later in development and contains only mycobiont hyphae, so it may be a means of acquiring new algal cells even later in life (Sanders and Lücking 2002). Similarly, in the study of *Cladonia* species it was found that the podetia can contain more and different species of photobionts than the base (older) part of the thallus, suggesting that during the formation of new structures the lichen may open a new path for expanding the photobiont diversity (Bačkor et al. 2010). There is probably also the possibility to acquire new photobionts in older thalli through fusion of two genetically different lichens. This phenomenon has been observed in *Parmotrema tinctorum*, and thallus lobe fusion has also been documented across other lichen genera (Ohmura et al. 2006, Armstrong 1984, Mansournia et al. 2012). It has been hypothesized that such structural integration may provide a potential route for the horizontal transfer of algal partners. Although *P. muralis* does not produce any specialized structures for photobiont uptake, its continuous growth via the formation of new lobes could facilitate similar interactions, potentially enabling the incorporation of nonnative photobionts over time.

Interestingly, the age of the lichen seems to influence the abundance of algae with different reproductive strategies. As the lichen grows older, the abundance of autosporine algae increases at the expense of zoosporine algae, which are much more abundant inside the young thalli. It should be noted that some of the smallest thalli included only zoosporine species. This could correlate with the tendency of the lichen to keep only species of algae that are easier to keep under control by the mycobiont partner. On the other hand, the zoospores could be advantageous at the beginning of lichen development, since it might be easier for the mycobiont hyphae to get into contact with algae that can be attracted from free-living guilds of algae, making the process of relichenization more effective (Slocum et al. 1980, Sanders 2005).

### Flexibility and specificity of photobiont selection

The hypothesis that young lichens are likely less specific in photobiont choice compared to adults has been mostly studied in parasitic lichens. For example, in the lichen *Disploschistes muscorum*, which overgrows other lichens of the genus *Cladonia*, young thalli contain the photobiont species identical to the host *Cladonia* lichen, i.e. *Asterochloris irregularis*. They steal it by penetrating the host’s cortex and entering the photobiont layer. However, as *D. muscorum* matures and forms a separate thallus, it exchanges the photobiont for another species, *T.showmanii*, which is likely more preferred at this later life stage (Friedl 1987). It seems to be a typical trait for this genus, although the roles can be interchangeable. For example, another species, *D. diacapsis*, has been proposed as a potential donor of compatible symbionts for various lichens and is able to associate with at least three different *Trebouxia* species, from which other lichenicolous lichens can select symbionts for incorporation into their own thalli (Moya et al. 2020 ). Similarly, in the lichen *Xanthoria parietina*, it was confirmed that the initial stages of the development can be formed with any available coccal green algae, not necessarily a specific species. These algae can even be obtained from soredia of other lichens and maintain this type of interaction for an extended period. This suggests that less advantageous algae might be detected within the thallus of young lichens. However, in later stages, the presence of a specific preferred algal species is required, as the change always occurs in the older thallus (Ott 1987, Moya et al. 2020).

Although in the end we were not able to confirm the hypothesis of less specific algal choice in early development for the lichen *P. muralis*, this opportunistic strategy could still be a mechanism present in other lichen species and merits further investigation. It has the potential to enable young lichens to rapidly establish symbiotic relationships with any available coccoid green algae from their environment, making it easier to create a symbiotic pair in a short period of time. However, as lichens mature, the persistence of multiple photobiont lineages probably offers resilience against environmental fluctuations, with dominant types prevailing under current conditions and others persisting at lower abundances. This reservoir of diversity could enable the lichen to adapt to environmental changes by modulating photobiont dominance (Casano et al. 2011, Muggia et al. 2014).

### Unexpected algal dominance and photobiont turnover

When focusing on data concerning *Trebouxia* dominance in our samples, some very interesting results emerged from the DNA metabarcoding analyses. In 10 samples, the genus *Trebouxia* was present at only ~2% to a maximum of 26% of the overall algal abundance within the thallus. This stands in contrast to previous studies, where *Trebouxia* has consistently been reported as the dominant photobiont in *P. muralis* (Guzow-Krzemińska 2006, Guzow-Krzemińska and Stocker-Wörgötter 2013, Muggia et al. 2013). In these cases, where *Trebouxia* was not dominant, the most abundant genera were either *Elliptochloris, Asterochloris*, or *Pseudochlorella*, and in one case, *Chloroidium*. This is an interesting finding, which also correlated with our Sanger sequencing data, where in two cases, we identified *Asterochloris* or *Pseudochlorella* as the dominant photobiont. We do not consider these taxa to be contaminants or epiphytes, as the most abundant algae detected are known frequent symbionts in lichen partnerships, rather than incidental or nonsymbiotic species. The thalli with the low amounts of *Trebouxia* algae were distributed across various thallus sizes, though most were from the largest/oldest thalli.

As this has never before been documented for *P. muralis*, we can only hypothesize why this occurs within the lichen thallus, as *Trebouxia* is generally considered the most favorable symbiont for this lichen species (Guzow-Krzemińska and Stocker-Wörgötter 2013 , Muggia et al. 2013). This notable variability in algal composition could be the result of the progressed age of the lichen thalli, possibly influenced by various degrading factors, such as herbivory, disease, overgrowth, or other stressors that mostly affect older lichens (but could also impact younger ones). These factors may lead to the exclusion or degradation of certain symbionts or promote a shift in preference toward non-*Trebouxia* taxa. For example, some species of the aforementioned genera have been documented to tolerate harsher or more variable conditions. Several *Asterochloris* species have been described from Antarctica, exhibiting cold, desiccation, and UV tolerance (Kim et al. 2020). Under these extreme conditions, a newly described *Asterochloris* species was even identified as the photobiont of *Sphaerophorus globosus*, a lichen generally known to associate with *Trebouxia* (Kim et al. 2017, Zhu et al. 2024). *Chloroidium* has demonstrated genetic adaptation to environmental stress (Mamut et al. 2025), and *Pseudochlorella pringsheimii* has shown heavy metal and salinity tolerance (Ismaiel et al. 2024). These findings not only underscore the adaptability of these photobiont genera but also illustrate how environmental pressures can reshape symbiotic pairings. We did not observe any consistent pattern across these samples, so the underlying cause could involve adaptive responses to unmeasured environmental factors, such as subtle variations in humidity, light exposure, or substrate chemistry, which may favor certain photobionts and alter composition (Armstrong et al. 2015b).

Lastly, the observed discrepancy could stem from technical issues in the sequencing process. Specifically, the use of highly specific green-algal or even *Trebouxia*-targeted primers may inadvertently bias amplification toward certain taxa, thereby underrepresenting or entirely missing others. In this study, we used primers, which successfully detected cases where *Trebouxia* was entirely absent. In two samples sequenced via the Sanger method, we identified *Pseudochlorella* and *Asterochloris* as the dominant photobionts. In contrast, previous Sanger-based studies of *P. muralis* have consistently reported only *Trebouxia*, likely due to the use of highly specific primers. Our DNA metabarcoding further identified thalli lacking dominant *Trebouxia* representation—an observation never previously documented. This discrepancy may stem from differences in primer specificity. The broader-spectrum primers used in our study likely enabled detection of taxa that were overlooked in previous research. In contrast, major studies on *P. muralis* have typically used photobiont-specific primers, even exclusively targeting *Trebouxia*. These include primers AL1500bf (Helms et al. 2001), ITS4M (Guzow-Krzemińska 2006), and nucSSU-1780–5′ (Piercey-Normore and DePriest 2001). Notably, some of these primers carry substitutions at the 3′ end, skewing amplification in favor of *Trebouxia* sequences even when this genus may not be dominant in the sample. These biases likely contributed to the limited diversity reported in earlier studies and highlight why our approach yielded a more comprehensive and nuanced view of the photobiont landscape in *P. muralis* (Guzow-Krzemińska 2006, Guzow-Krzemińska and Stocker-Wörgötter 2013, Muggia et al. 2013).

Finally, it is important to mention that the depth of sequencing is crucial for studying photobiont diversity and adaptation. When focusing exclusively on dominant algal taxa via Sanger sequencing, photobiont diversity often appears low, typically represented by *Trebouxia* species from clade A. However, DNA metabarcoding reveals a far richer spectrum of diversity spanning three *Trebouxia* clades, each with distinct ecological preferences. Moreover, it allows detection of other algal genera in low abundances which, although sparsely represented, still contribute meaningfully to the complex phycobiome within the thallus.

## Conclusion

The diversity of the photobionts within a lichen thallus can vary significantly, even within a single lichen species, as demonstrated in this study on *P. muralis*. The age of lichen thalli has a notable impact on this composition. Older thalli exhibit a greater overall diversity of algal species, while younger thalli exhibit more limited diversity. Notably, *P. muralis* does not always display *Trebouxia* as the dominant photobiont genus. Exceptions appear to arise in response to environmental shifts or changes in the physiological state of the thallus, which may favor alternative symbionts. Currently, there are only a few studies addressing photobiont diversity in relation to the ontogenetic stage of lichens. It is essential to continue research in this area, as the results can vary greatly among different lichens.

## Supplementary Material

fiaf096_Supplemental_Files

## Data Availability

The genetic data reported in this paper have been deposited in the National Center for Biotechnology Information (NCBI) Short Read Archive under the BioProject: PRJNA1230568. ITS rRNA sequences have been deposited in the NCBI, GenBank: PV406848–PV406935. Multiple alignments of ITS rRNA sequences are freely available on Mendeley Data: https://doi.org/10.17632/jrcxghspjw.1.
